# Supplementary material for: End Sequence Analysis Toolkit (ESAT) expands the extractable information from single-cell RNA-seq data
Source: Genome Res. 2016 Oct;26(10):1397–410. doi: 10.1101/gr.207902.116 (PMC5052061; doi:10.1101/gr.207902.116)
Supplement: Supplemental Material [file supp_gr.207902.116_Supp_Materials.tar.gz › ESAT-esat_v0.1/doc/html/classbroad_1_1pda_1_1samtools_1_1_filter_paired_reads_by_m_a_q.html]

ESAT: broad.pda.samtools.FilterPairedReadsByMAQ Class Reference


ESAT


- Main Page
- Related Pages
- Packages
- Classes
- Files

- Class List
- Class Index
- Class Hierarchy
- Class Members

All Classes Namespaces Files Functions Variables Enumerator Pages

- broad
- pda
- samtools
- FilterPairedReadsByMAQ

Static Public Member Functions |
Public Attributes |
Protected Member Functions |
List of all members

broad.pda.samtools.FilterPairedReadsByMAQ Class Reference

Inheritance diagram for broad.pda.samtools.FilterPairedReadsByMAQ:

[legend]

Collaboration diagram for broad.pda.samtools.FilterPairedReadsByMAQ:

[legend]

|  |  |
| --- | --- |
| Static Public Member Functions | |
| static void | main (final String[] args) |
|  | |

|  |  |
| --- | --- |
| Public Attributes | |
| File | INPUT |
|  | |
| Integer | MINIMUM\_MAPPING\_QUALITY = 30 |
|  | |
| File | OUTPUT |
|  | |
| Boolean | REMOVE\_MATE = false |
|  | |
| File | METRICS =null |
|  | |
| SAMFileHeader.SortOrder | SORT\_ORDER = SAMFileHeader.SortOrder.coordinate |
|  | |

|  |  |
| --- | --- |
| Protected Member Functions | |
| int | doWork () |
|  | |

## Detailed Description

Author
:   engreitz Remove reads that do not map uniquely to the genome. If a read's mate is removed this way, mark that read as unpaired.

## Member Function Documentation

|  |  |  |  |  |
| --- | --- | --- | --- | --- |
| int broad.pda.samtools.FilterPairedReadsByMAQ.doWork | ( |  | ) |  |

protected

|  |  |  |  |  |  |
| --- | --- | --- | --- | --- | --- |
| static void broad.pda.samtools.FilterPairedReadsByMAQ.main | ( | final String[] | *args* | ) |  |

static

Stock main method.

Parameters
:   |  |  |
    | --- | --- |
    | args | main arguments |

## Member Data Documentation

File broad.pda.samtools.FilterPairedReadsByMAQ.INPUT

File broad.pda.samtools.FilterPairedReadsByMAQ.METRICS =null

Integer broad.pda.samtools.FilterPairedReadsByMAQ.MINIMUM\_MAPPING\_QUALITY = 30

File broad.pda.samtools.FilterPairedReadsByMAQ.OUTPUT

Boolean broad.pda.samtools.FilterPairedReadsByMAQ.REMOVE\_MATE = false

SAMFileHeader.SortOrder broad.pda.samtools.FilterPairedReadsByMAQ.SORT\_ORDER = SAMFileHeader.SortOrder.coordinate

---

The documentation for this class was generated from the following file:

- java/broad/pda/samtools/FilterPairedReadsByMAQ.java


---

Generated on Fri Aug 15 2014 15:11:01 for ESAT by  

 1.8.7
